# The glycolysis-lactylation axis: a metabolic-epigenetic driver of immunosuppression and therapy resistance in cancer

**DOI:** 10.3389/fimmu.2026.1772701

**Published:** 2026-04-14

**Authors:** Simin Tan, Weijia Zhang, Yuqing Ding, Jinlin Liu, Min Zhu, Xuefeng Luo, Jun Cai, Hai Zeng

**Affiliations:** Department of Oncology, The First Affiliated Hospital of Yangtze University, Jingzhou, Hubei, China

**Keywords:** glycolytic reprogramming, immune evasion, immunotherapy resistance, lactylation, metabolic-epigenetic, tumor microenvironment (TME)

## Abstract

**Background:**

The immunosuppressive tumor microenvironment (TME) is a major barrier to the efficacy of cancer immunotherapy. Tumor metabolic reprogramming, particularly aerobic glycolysis (the Warburg effect), drives lactate accumulation in the TIME. Beyond fueling tumor growth, lactate-derived lysine lactylation (Kla) has emerged as a pivotal epigenetic and post-translational modifier, directly coupling metabolic activity to the regulation of immune cell function and tumor cell resilience.

**Main body:**

This review synthesizes current evidence to delineate how the glycolysis-lactylation axis orchestrates a multi-faceted immunosuppressive program and confers broad therapy resistance. We detail its mechanisms in: (1) Inhibiting antitumor immunity by driving M2 macrophage polarization, enhancing regulatory T cell (Treg) function, and promoting CD8^+^ T cell exhaustion; (2) Enhancing intrinsic tumor cell resistance through lactylation-mediated DNA damage repair and stemness maintenance; and (3) Directly undermining immunotherapy, notably by stabilizing programmed cell death 1 ligand 1 (PD-L1). We critically evaluate emerging therapeutic strategies that target this axis, including inhibitors of glycolytic enzymes, lactate transporters (MCTs), and lactylation writers/erasers, and their potential to synergize with established immunotherapies.

**Conclusions:**

Targeting the lactate-lactylation signaling hub represents a promising metabolic-epigenetic strategy to dismantle tumor-driven immunosuppression and overcome therapeutic resistance, particularly resistance to immunotherapy. Although a substantial body of preclinical evidence, ranging from cancer cell line models to patient-derived xenografts, supporting the potential of targeting this axis, its clinical translation remains hindered by a gap in the evidence hierarchy, necessitating further validation through prospective clinical trials.

## 1 Introduction

Metabolic reprogramming is an established hallmark of cancer, with the abnormal activation of aerobic glycolysis, the Warburg effect being particularly prevalent (1). This metabolic shift results in substantial lactate production and secretion, acidifying the TME and providing biosynthetic precursors for rapidly proliferating cells (2). Beyond its role as a metabolic byproduct, lactate is now recognized as a key oncometabolite and signaling molecule. It is shuttled across cell membranes via monocarboxylate transporters (MCTs) and contributes to critical cancer-promoting processes such as angiogenesis and, most importantly, immunomodulation (3, 4).

A groundbreaking discovery by Zhang et al. revealed that lactate serves as a precursor for a novel post-translational modification (PTM): lysine lactylation (Kla) (5). This reversible modification occurs on both histone and non-histone proteins, directly linking the intracellular metabolic flux to epigenetic reprogramming and altering protein function (6). Consequently, lactylation has been shown to regulate diverse tumorigenic processes, including proliferation, invasion, and immune escape.

Immunotherapy, especially immune checkpoint blockade, has revolutionized cancer treatment, but its efficacy is often limited by primary or acquired resistance. A significant contributor to this resistance is the immunosuppressive TME, where metabolites like lactate play a detrimental role (7, 8). Emerging evidence positions the glycolysis-lactylation axis as a central integrator of multiple resistance mechanisms. Lactylation can directly silence anti-tumor immune responses, enhance tumor cell DNA repair after genotoxic stress, and promote a stem-like, resilient phenotype (8, 9). However, a comprehensive synthesis focusing on how this axis specifically drives immunosuppression and immunotherapy failure is lacking.

In this review, we systematically dissect the glycolysis-lactylation axis as a central metabolic-epigenetic driver orchestrating broad-spectrum therapeutic resistance. Moving beyond existing summaries of lactylation mechanisms, we adopt a clinically oriented framework that integrates resistance mechanisms across radiotherapy, chemotherapy, targeted therapy, and immunotherapy, with particular emphasis on its pivotal role as a metabolic-immune hub that coordinately remodels the TME. By synthesizing these multilayered insights, we aim to provide a critical translational perspective, evaluating both the therapeutic potential and existing gaps in targeting this axis to establish a theoretical foundation for developing rational combination strategies that overcome therapy resistance in cancer.

## 2 Glycolytic metabolic reprogramming: the metabolic basis of tumor therapy resistance

Resistance to tumor treatment is the main challenge in clinical practice. The Warburg effect not only provides energy for tumor cells, but also drives resistance to radiotherapy, chemotherapy and targeted therapy at multiple levels (9).

### 2.1 Promotion of cell survival and proliferation

Key glycolytic enzymes such as hexokinase 2 (HK2) have been confirmed to localize to the mitochondrial outer membrane in multiple cancer cell lines, where they interfere with apoptotic signal transduction by competitively inhibiting Bax binding to the voltage-dependent anion channel, thereby promoting cell survival (10). This metabolic advantage is further reinforced by epigenetic mechanisms that sustain glycolytic gene expression. For instance, in colorectal cancer (CRC) cell lines and mouse xenotransplantation models, Methyltransferase-like 3 (METTL3) can stabilize its expression by driving m6A modification of HK2 and glucose transporter (GLUT1, GLUT2, GLUT3, GLUT4) mRNA, thereby enhancing glycolysis and promoting tumor progression (11). This regulation leads to the sustained high expression of key glycolytic enzymes, including HK2, Phosphofructokinase-1, platelet isoform, Aldolase A (ALDOA), and Pyruvate dehydrogenase kinase 1, which collectively serve dual functions: providing ample ATP and biosynthetic precursors to fuel rapid proliferation, and maintaining the reduced form of nicotinamide adenine dinucleotide phosphate (NADPH)/reduced glutathione antioxidant system (12).The functional importance of this glycolytic program is underscored by loss-of-function studies: genetic or pharmacological inhibition of these glycolytic enzymes significantly impairs tumor cell growth, indicating that disrupting glycolytic homeostasis can trigger cancer cell death (13). Collectively, these interconnected mechanisms from direct anti-apoptotic functions of HK2 to epigenetic reinforcement of glycolytic capacity establish a metabolic foundation for therapy resistance by enhancing both cell survival and stress tolerance.

### 2.2 Mediation of radiotherapy resistance

Radiotherapy exerts its antitumor effects primarily by inducing DNA double-strand breaks. However, tumor cells counteract this through metabolic reprogramming, particularly the shunting of glycolytic intermediates into the pentose phosphate pathway (PPP) and serine synthesis pathway (SSP) (14, 15). A central node in this adaptive response is pyruvate kinase M2 (PKM2): radiation-induced inhibition of PKM2 activity diverts metabolic flux away from glycolysis toward PPP and SSP, thereby generating abundant NADPH, glutathione, and nucleotide precursors that collectively enhance oxidative stress resistance and provide substrates for DNA damage repair (16, 17). This PPP flux is further potentiated through two synergistic mechanisms. At the transcriptional level, radiation-induced oxidative stress stabilizes nuclear factor erythroid 2-related factor 2 (NRF2), which translocates to the nucleus and upregulates the PPP rate-limiting enzyme glucose-6-phosphate dehydrogenase (G6PD) (16, 17). Concurrently, the DNA damage response kinase ATM directly phosphorylates and activates G6PD, enhancing its enzymatic activity through post-translational modification (15, 18). Together, these transcriptional and post-translational controls ensure sustained production of reducing equivalents for antioxidant defense and DNA repair.

These metabolic adaptations are amplified by the tumor microenvironment. Hypoxia stabilizes HIF-1α, which transcriptionally upregulates GLUT1 and glycolytic enzymes such as lactate dehydrogenase A (LDHA), establishing a positive feedback loop that exacerbates radiation resistance (18). Moreover, cancer stem cells (CSCs) exhibit heightened reliance on this metabolic program: they maintain their self-renewal capacity and radio resistance through dynamic inhibiting PKM2 conformation (favoring the less active dimeric form to support PPP flux) and enhanced membrane translocation of GLUT3 to secure glucose uptake under stress (1).

### 2.3 Driving chemotherapy resistance

Enhanced glycolytic activity and increased lactate production are consistently observed in chemotherapy-resistant cancer cell lines (19). This metabolic shift contributes to chemoresistance through multiple interconnected mechanisms that operate at both the tumor cell-intrinsic and microenvironmental levels. Lactate-mediated acidification of the tumor microenvironment alters the ionization state of weakly basic chemotherapeutic agents, thereby impairing their intracellular accumulation (6). Concurrently, glycolysis-generated ATP fuels ATP-binding cassette (ABC) transporters including MDR1, MRP1, and BCRP, whose upregulated expression and enhanced efflux activity actively pump chemotherapeutic drugs out of cancer cells, the mechanism has been verified by pharmacological inhibition experiments in drug-resistant cell models (20, 21). Beyond these direct effects, upregulation of the glycolytic enzyme aldolase A (ALDOA) simultaneously enhances glycolytic flux and PPP activity, promoting nucleotide synthesis and DNA repair to counteract chemotherapy-induced DNA damage (2).

Lactate-driven activation of cancer-associated fibroblasts impairs drug penetration into the tumor microenvironment. More importantly, lactate directly suppresses CD8^+^ T cell proliferation and effector cytokine production (e.g., IFN-γ, granzyme B), inducing T cell apoptosis, and impairing T cell receptor signaling through microenvironmental acidification (4, 22, 23). Collectively, these mechanisms drive tumor-infiltrating CD8^+^ T cells into a state of functional exhaustion, thereby crippling antitumor immune responses and indirectly fostering drug resistance. Through this dual-layer strategy, enhancing tumor cell-intrinsic drug tolerance while simultaneously sculpting an immunosuppressive and drug-excluding microenvironment glycolytic reprogramming establishes a multifaceted barrier to effective chemotherapy.

### 2.4 Mediation of targeted therapy resistance

Under the pressure of the selection of targeted drugs, tumor cells evade treatment through metabolic reprogramming (19). In HER2-positive breast cancer, resistance to trastuzumab is closely associated with activation of the HSF1–LDHA signaling axis, which drives a metabolic shift from oxidative phosphorylation (OXPHOS) toward glycolysis and is concomitantly linked to the acquisition of stem cell-like properties, a phenotype that can be reversed by glycolysis inhibitors (24). Beyond such signaling-driven metabolic adaptation, epigenetic regulation of glucose metabolism represents an additional layer of resistance. For instance, ALKBH5-mediated m6A demethylation of GLUT4 mRNA enhances glycolytic capacity by stabilizing GLUT4 expression, thereby contributing to targeted therapy resistance (25). Together, these signal and epigenetic-driven metabolic reprogramming mechanisms illustrate the multi-layered adaptive strategies through which tumor cells withstand targeted therapeutic pressure.

Notably, this metabolic adaptation often coexists with and may be reinforced by the reactivation of oncogenic signaling pathways, which provide complementary survival signals under therapeutic pressure. For example, B-Raf proto-oncogene, serine/threonine kinase (BRAF) inhibitor drug-resistant cells sustain proliferation through upregulated OXPHOS or enhanced glycolysis (26, 27). In microsatellite stable colorectal cancer, aberrant activation of the WNT/β-catenin pathway represents a distinct immune resistance mechanism, disrupting anti-tumor immunity by suppressing CD8^+^ T cell infiltration, downregulating MHC class I expression, and inhibiting interferon-γ (IFN-γ) signaling (28). These examples illustrate how metabolic and signaling adaptations converge to drive a signal-metabolism dual-track escape mode, enabling tumors to simultaneously resist direct cytotoxic effects and evade immune surveillance.

## 3 Lactylation modification: a critical bridge connecting glycolysis to tumor therapy resistance

**Figure 1:**
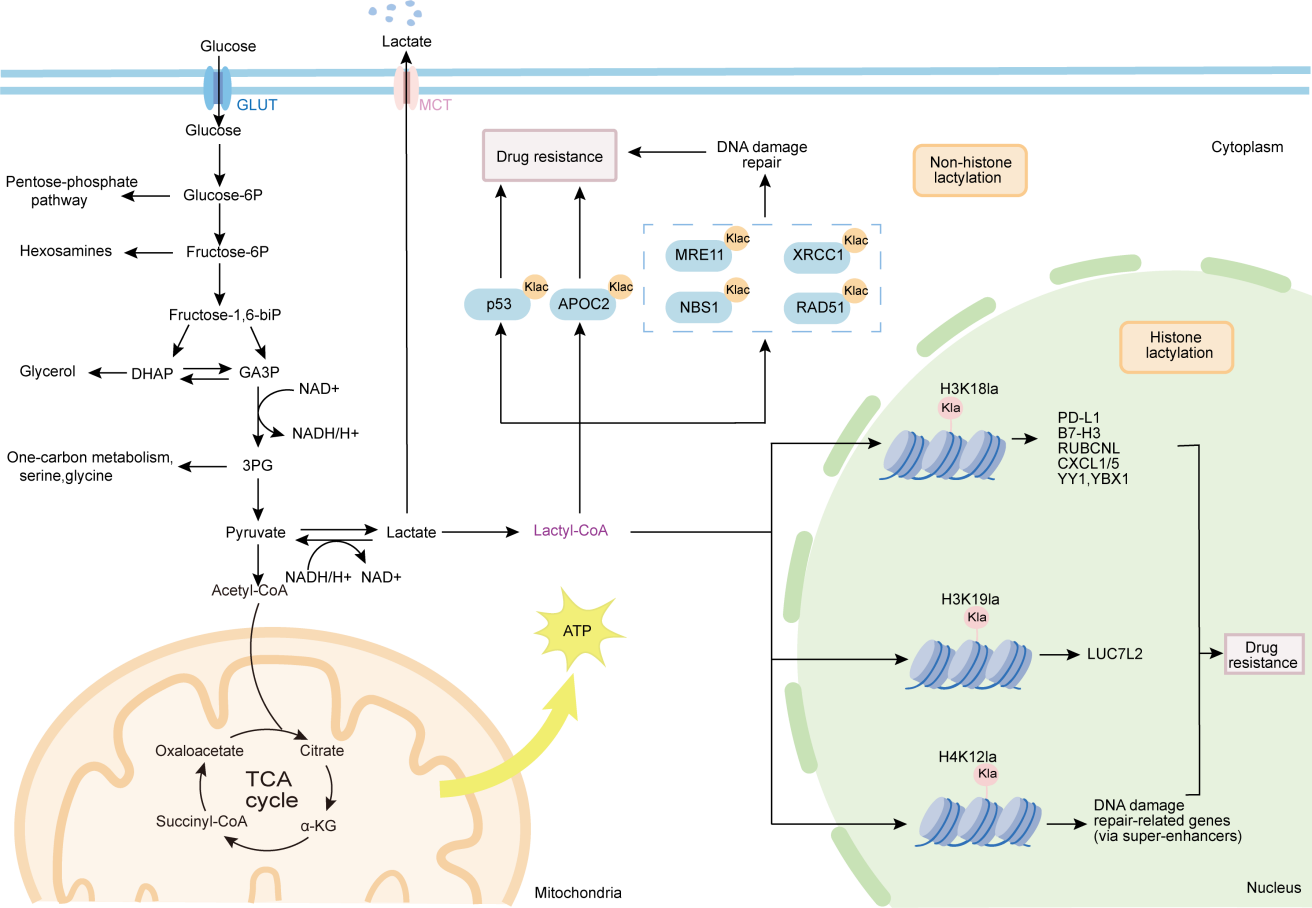
The glycolysis lactylation axis drives the core mechanism of tumor multidrug resistance. Lactic acid derived from Warburg effect promotes resistance through lactylation modification, including histone lactylation (activating ABC transporters, immunomodulatory molecules and splicing factors) and non-histone lactylation (enhancing DNA repair and inhibiting apoptosis). ABC, ATP binding box; CXCL, C-X-C motif chemokine.

Here we provide a schematic overview of the glycolysis-lactylation axis and its contribution to multi-modal therapy resistance. As depicted, lactate derived from the Warburg effect serves not only as a metabolic byproduct but also as a precursor for protein lactylation, which subsequently establishes a self-amplifying feedforward loop through transcriptional upregulation of glycolytic enzymes such as LDHA and HK2 (**Figure 1**). The lactate accumulation driven by the Warburg effect not only alters the acid-base balance of the tumor microenvironment, but more importantly, serves as a precursor initiating protein lactylation modification (29). This emerging PTM constitutes a critical pathway linking metabolic abnormalities to epigenetic remodeling, thereby tightly coupling glycolysis and lactylation into a self-reinforcing feedforward regulatory circuit. Specifically, glycolysis provides the substrate for lactylation, while lactylation in turn transcriptionally upregulates key glycolytic enzymes such as LDHA and HK2. This reciprocal reinforcement sustains the hypermetabolic state of tumor cells and profoundly contributes to therapeutic resistance (30–33).

### 3.1 The distinct positioning of lactylation within the landscape of metabolism-derived PTMs

Tumor metabolic reprogramming drives diverse post-translational modifications derived from metabolic intermediates, including acetylation (acetyl-CoA-dependent), succinylation (succinyl-CoA-dependent), crotonylation (crotonyl-CoA-dependent), and lactylation (lactate-dependent) (34–36). These acylation modifications collectively constitute a complex molecular network that connects cellular metabolic states with epigenetic regulation and protein function (37). Notably, they share key regulatory enzyme systems: p300/CBP functioning as a broad-spectrum acyltransferase catalyzes lysine acetylation, succinylation, crotonylation, and lactylation, while class I histone deacetylases (HDAC1–3) and sirtuins (particularly SIRT1–3) exhibit extensive deacylase activity that reverses these modifications (7, 38–40).

This shared enzymatic machinery raises a central question: in the context of tumor therapeutic resistance, why focus on lactylation rather than other metabolism-driven acylation modifications? As the most extensively studied acylation modification, acetylation has paved the way for therapeutic interventions such as HDAC inhibitors (HDACi), which target global acylation networks and, given that HDACs also function as erasers of lactylation, inherently modulate the lactylation landscape. However, the uniqueness of lactylation is first manifested in its direct and exclusive coupling with the Warburg effect (41). Unlike acetyl-CoA, which can be derived from multiple pathways including glycolysis, fatty acid β-oxidation, branched-chain amino acid catabolism, and glutamine metabolism, lactate is the dedicated end-product of aerobic glycolysis (35, 42). Consequently, the significantly elevated lactate levels in tumor tissues—5- to 20-fold higher than in normal tissues, constitute a core metabolic feature that directly drives lactylation, whereas acetylation and succinylation are subject to cross-regulation by diverse metabolic inputs, rendering them relatively less specific as indicators of particular metabolic states (7, 43). More importantly, this coupling establishes a metabolic-epigenetic positive feedback loop: lactate upregulates the transcriptional expression of key glycolytic enzymes such as LDHA and PKM2 through histone lactylation (e.g., H3K18la, H4K12la), thereby consolidating high glycolytic flux into a durable epigenetic state, which a characteristic not shared by acetylation or succinylation modifications (18, 44). This distinctive metabolic origin underscores the importance of investigating lactylation dynamics, particularly under therapeutic pressure such as HDACi treatment, offering a promising avenue for future clinical translation targeting tumor metabolism.

Beyond metabolic specificity, lactylation exhibits unique regulatory breadth by integrating multidimensional resistance networks. Functioning as a master orchestrator of the TME, lactylation simultaneously drives M2 macrophage polarization, maintains Treg functional stability, and induces CD8^+^ T cell exhaustion (5, 45, 46). While acetylation also regulates immune cell differentiation, lactylation occupies a more prominent position in tumor immunity. More critically, lactylation concurrently orchestrates multiple resistance mechanisms: it enhances DNA repair capacity by inducing lactylation of key repair proteins such as X-ray repair cross-complementing protein 1 (XRCC1), Meiotic recombination 11 homolog 1 (MRE11), and RAD51 recombinase (RAD51), maintains cancer stem cell properties through lactylation of β-catenin and serine hydroxy methyltransferase 2 (SHMT2), and regulates immune checkpoint molecule expression via histone lactylation (47–50). This multifaceted mode of action establishes lactylation as a common hub for resistance to radiotherapy, chemotherapy, targeted therapy, and immunotherapy, setting it apart from acetylation or succinylation, which have more focused regulatory scopes.

The unique kinetic characteristics of lactylation, coupled with its distinct potential for clinical translation, further strengthen its therapeutic relevance. Zhang et al. demonstrated that lactylation exhibits fundamentally different kinetics compared to acetylation: histone acetylation peaks rapidly within hours, whereas lactylation accumulates gradually over 24 hours, indicating its unique function as an integrator of sustained metabolic stress (5). More importantly, the gene sets targeted by lactylation and the resulting transcriptional consequences are distinct from those of acetylation, a finding that underscores the unique role of lactylation in converting persistent metabolic alterations into durable epigenetic reprogramming (5). Furthermore, the close association of lactylation with the PD-1/PD-L1 axis, tumor angiogenesis, and chemotherapy resistance positions it as an ideal molecular target for combination strategies integrating metabolic interventions such as LDH inhibitors with immunotherapy (51, 52).

In summary, the fundamental rationale for focusing on lactylation rather than other metabolic modifications lies in its role as a direct molecular bridge between the Warburg effect and therapy resistance. Its intervention node located downstream of this bridge and having tumor relative specificity. Furthermore, its intervention nodes (LDHA, MCT1/4, p300) are situated downstream of this bridge and exhibit relative tumor specificity. It is precisely the specificity of its metabolic origin, the breadth of its functional integration, and the accessibility of its therapeutic targeting that collectively establish the unique position of lactylation within the landscape of tumor metabolism and epigenetic research.

### 3.2 Molecular mechanisms of lactylation modification

The dynamic equilibrium of lactylation modification is coordinately maintained by three core classes of regulatory elements: writer enzymes that catalyze the addition of lactyl groups, eraser enzymes that mediate their removal, and readers that recognize modification signals and execute downstream functions. Collectively, these three classes of regulators determine the intensity, duration, and functional output of lactylation modifications, constituting the molecular interface that links cellular metabolic states with phenotypic regulation. This framework thereby establishes a tight coupling between metabolic reprogramming and epigenetic remodeling.

#### 3.2.1 Writer enzymes

Writer enzymes are responsible for catalyzing the covalent attachment of a lactyl group to lysine residues of proteins (53). These enzymes recognize lactate or its activated form and promote the modification reaction with target proteins (54). Through *in vitro* biochemical assays and cellular functional validation, the histone acetyltransferase p300/CBP has been identified as the most extensively characterized lactylation writer to date, capable of catalyzing lactylation modifications at sites such as H3K18 and H3K9 (55). Furthermore, other enzymes such as HBO1, KAT8, and the newly discovered alanyl-tRNA synthetase (AARS), which operates via a non-canonical, Lactyl-CoA-independent pathway, have also been confirmed to possess lactyltransferase activity (38). In CRC cell lines, KAT8 was demonstrated to possess lactyltransferase activity, catalyzing the lactylation of Eukaryotic translation elongation factor 1 alpha 2 (eEF1A2) (56). More importantly, through metabolite binding assays and tumor cell models, AARS1 was identified as a lactate sensor and lactyltransferase that directly catalyzes the lactylation of p53, thereby playing pivotal roles in processes including translation elongation and tumor suppression (57).

However, the substrate selectivity of different writers and their cross-regulatory mechanisms with acetylation modifications remain key foci for future research (58). More importantly, current related studies are largely confined to preclinical models, lacking dose-response relationship data and intervention endpoint data from clinical samples, which directly hinders their clinical translation progress.

#### 3.2.2 Eraser enzymes

Eraser enzymes are responsible for removing lactylation modifications, enabling dynamic reversal of this modification and thereby regulating the functions of target proteins and downstream gene expression (53). Several deacetylases, including Class I histone deacetylases HDAC1–3 and Class III deacetylase SIRT1,3, have been demonstrated to possess delactylation activity; however, their catalytic efficiency and selectivity for substrate stereo configuration vary significantly (39, 59, 60). For instance, in pancreatic ductal adenocarcinoma (PDAC), histone deacetylases 2 has been shown to specifically remove H3K18la marks, illustrating the tissue and substrate specificity of eraser enzyme function (30).

Currently, research on lactylation erasers remains primarily focused on histone modifications. The system of delactylases for non-histone proteins (e.g., metabolic enzymes, DNA repair factors) has not been fully elucidated; their substrate recognition mechanisms and pathophysiological functions will be important directions for future research.

#### 3.2.3 Reader proteins

Reader proteins can specifically recognize and bind lactyl-lysine-modified proteins or functional domains (54). Research in this area is still in its infancy. Currently known acetylation reading modules may cross-identify lactic acid modifications, but only a few proteins such as Brg1 have been preliminarily confirmed as specific readers of H3K18la (61). The key to the complete disclosure of the lactic acid regulation network is to systematically identify and verify the highly specific lactic acid reader and analyze its functions.

### 3.3 Lactylation modification and tumor drug resistance

The multifaceted roles of lactylation in driving resistance to radiotherapy, chemotherapy, targeted therapy, and immunotherapy are depicted in **Figure 1**. Lactylation modification serves as a key driver of chemoresistance, extensively participating in the adaptive resistance of tumor cells to chemotherapeutic agents.

#### 3.3.1 Radiotherapy resistance

Lactylation directly drives radio resistance by enhancing DNA damage repair capability (62). Lactylation at the K247 residue of XRCC1 promotes its nuclear translocation and significantly enhances the DNA repair capacity of glioblastoma cells, thereby inducing radio resistance. This mechanism has been validated both *in vitro* in cell lines and *in vivo* in orthotopic mouse models, where tumors expressing a lactylation-deficient XRCC1 mutant exhibited increased sensitivity to radiotherapy (49). Furthermore, histone lactylation marks such as H3K18la have been shown in cancer cells to globally upregulate the transcription of key homologous recombination (HR) repair genes including breast cancer susceptibility protein 1 and RAD51, thereby systematically enhancing double-strand break repair capacity (48). Concurrently, lactylation suppresses the transcriptional activity of p53 and attenuates radiation-induced apoptosis in various tumor models (63, 64). These mechanisms collectively establish a positive feedback cycle of radiation-hypoxia-lactate accumulation-repair enhancement, ultimately contributing to radiotherapy failure.

#### 3.3.2 Chemotherapy resistance

These mechanisms reveal that lactylation is involved in radiation resistance by enhancing DNA damage repair. In addition to radiotherapy, chemotherapy, as another type of DNA damage treatment, is also profoundly affected by lactylation modification, but its mechanism is more diverse, involving transcription regulation, drug efflux and DNA repair.

Histone lactylation can promote the expression of drug resistance-related genes by altering chromatin states. For instance, studies in CRC cell lines and patient-derived xenografts have demonstrated that H3K18la activates the autophagy gene Rubicon like autophagy enhancer (RUBCNL), conferring resistance to bevacizumab, whereas in glioblastoma cell lines and intracranial xenograft models, H3K9la upregulates LUC7 like 2, pre-mRNA splicing factor, resulting in loss of mutl homolog 1 (MLH1) expression and ultimately leading to temozolomide resistance (65, 66). In ovarian cancer cells and patient-derived organoids, H4K12la recruits MYC to activate super-enhancers, augmenting DNA damage repair capacity and inducing resistance to the Poly (ADP-ribose) polymerase (PARP) inhibitor niraparib (67). Furthermore, downregulation of structural maintenance of chromosomes protein 4 (SMC4) induces a dormancy-like state in CRC cells, accompanied by increased lactate production, which subsequently upregulates ABC transporter expression via the histone lactylation pathway, enhancing drug efflux capability (68). Similarly, in cisplatin-resistant bladder cancer cells, lactate accumulation driven by glycolytic activation elevates H3K18la levels; this modification activates transcription factors YY1 and YBX1, leading to the upregulation of cisplatin efflux pumps and eventual chemotherapy failure (69). In oral squamous cell carcinoma, histone lactylation (H3K18la and H4K12la) upregulates the transcription of Basal cell adhesion molecule (BCAM), which promotes resistance to cisplatin and other chemotherapy drugs by enhancing the survival signal mediated by tumor cell adhesion. Huang et al. further demonstrated through functional experiments that BCAM knockout can restore chemotherapy sensitivity (70).

Lactylation modification drives resistance by directly affecting the activity of key DNA repair proteins, thereby enhancing damage repair capacity. Studies in gastric cancer cell lines and organoids have revealed that lactylation of Nijmegen breakage syndrome 1 (NBS1) K388la and MRE11 K673la enhances DNA end resection by promoting MRN complex assembly, resulting in a more than two-fold increase in HR repair efficiency, thereby driving tumor cell resistance to platinum-based agents and PARP inhibitors (47, 48). Furthermore, lactylation at lysine residues K73 and K40 of RAD51 enhances its function in HR repair (47, 71). Functional studies in patient-derived xenograft (PDX) models and organoids have confirmed that inhibiting CBP or LDH to reduce MRE11 lactylation effectively impairs HR repair capacity, thereby restoring cellular sensitivity to chemotherapy agents and PARP inhibitors (48).

#### 3.3.3 Targeted therapy resistance

Unlike chemotherapeutic agents that directly damage DNA, targeted therapies act upon specific oncogenic drivers. Lactylation modification also participates in this resistance paradigm. Lactylation modification systematically reshapes the tumor immune microenvironment through a multicellular, multi-target regulatory network. Its modulation of immune cell functions is primarily manifested in three key populations: functional exhaustion of effector CD8^+^ T cells, expansion and enhanced functionality of regulatory T cells (Tregs), and polarization of tumor-associated macrophages toward the M2 phenotype (5, 22, 72). Collectively, these three elements establish the lactylation-driven immunosuppressive landscape, the specific mechanisms of which are as detailed below. Luo et al. demonstrated that lactylation of HIF1α enhances its protein stability and transcriptional activity, leading to direct upregulation of KIAA1199 in prostate cancer cells (52). Activation of the HIF1α–KIAA1199 axis subsequently promotes endothelial-like transdifferentiation and vasculogenic mimicry, establishing an alternative tumor blood supply that sustains tumor cell survival under enzalutamide treatment and ultimately confers therapeutic resistance (52). Furthermore, lactylation modification can synergistically promote the multidrug resistance phenotype by upregulating factors such as the long non-coding RNA LINC00152, the methyltransferase METTL3, and the potassium channel protein Kcnk6 (73–75). Notably, in preclinical models of Bevacizumab resistance, combined inhibition of histone lactylation and the autophagy pathway synergistically enhanced the antitumor efficacy of Bevacizumab, indicating that targeting the lactylation process is a potential synergistic strategy for reversing targeted therapy resistance (65). Collectively, these findings reveal that lactylation promotes therapeutic resistance through multiple convergent mechanisms, ranging from metabolic reprogramming and vasculogenic mimicry to the upregulation of diverse resistance-associated factors, underscoring its emerging role as a central node in adaptive resistance networks.

#### 3.3.4 Immune microenvironment remodeling and immunotherapy resistance

Resistance to targeted therapy primarily involves cell-intrinsic signaling adaptation within tumor cells, whereas resistance to immunotherapy entails more complex intercellular crosstalk. At this level, lactylation modification exerts its critical role by remodeling immune cell functions within the tumor microenvironment. In non−small cell lung cancer (NSCLC), accumulating evidence highlights lactylation as a pivotal epigenetic mechanism driving immune evasion. Notably, H3K18la has been implicated in multiple immune−regulatory pathways: it directly upregulates PD−L1 expression via the POM121/MYC axis on tumor cells, and independently activates transcription of the chemokines CXCL1 and CXCL5, thereby promoting the recruitment of myeloid−derived suppressor cells (MDSCs) into the tumor microenvironment, MDSC accumulation, in turn, suppresses CD8^+^ T cell cytotoxicity and contributes to resistance against ICB (76, 77). Beyond tumor−intrinsic effects, lactylation also modulates immune cell function directly, for example, H3K18la upregulates the immunosuppressive molecule B7−H3 in CD8^+^ T cells, inducing T cell exhaustion (45, 78, 79). Collectively, these findings illustrate that lactylation orchestrates immune evasion at multiple levels, through tumor cells, the chemokine milieu, and effector T cells, underscoring its potential as a therapeutic target to overcome immunotherapy resistance in NSCLC. Beyond tumor cell-intrinsic and T cell-mediated effects, lactylation extensively reshapes the TME through multiple cellular targets. Lactylation drives macrophage polarization toward the M2 phenotype and upregulates key regulators such as METTL3 in myeloid cells, thereby sustaining the activation of immunosuppressive signaling pathways including JAK-STAT (75, 80). Concurrently, lactate directly catalyzes K72 lactylation of the cytoskeletal protein MOESIN in Tregs, enhancing its interaction with TGF-β receptor I and subsequently activating SMAD3 signaling (45, 46). This modification promotes Treg expansion and functional maintenance, further reinforcing the immunosuppressive milieu. Collectively, these findings underscore the multifaceted nature of metabolic-immune crosstalk, wherein lactylation orchestrates immune evasion through parallel yet interconnected mechanisms involving tumor cells, myeloid cells, and Tregs. This complexity not only highlights the challenges in targeting the lactylation pathway but also opens new avenues for developing rational combination immunotherapies.

#### 3.3.5 Epigenetic control and cancer stem cell maintenance

The aforementioned mechanisms elucidate how lactylation influences differentiated tumor cells and their microenvironment. More fundamentally, lactylation also participates in maintaining the self-renewal capacity of CSCs, thereby seeding the potential for long-term tumor survival and recurrence. Lactylation contributes to the maintenance of cancer stem cell (CSC) properties through multiple interconnected mechanisms, ranging from metabolic reprogramming to direct modulation of core stemness pathways. In glioma stem cells, lactylation of polypyrimidine tract-binding protein 1 (PTBP1) at lysine k436 enhances its RNA-binding capacity and protein stability, thereby promoting its binding to and stabilization of mRNAs encoding key glycolytic enzymes such as 6-phosphofructo-2-kinase/fructose-2,6-bisphosphatase 4, which subsequently upregulates glycolytic flux (81). The resulting accumulation of lactate further drives lactylation of PTBP1 and other substrates, establishing a positive feedback loop in which lactylation potentiates glycolysis and glycolysis reinforces lactylation. This self-amplifying circuit plays a central role in sustaining the metabolic adaptability and self-renewal capacity of glioma stem cells (81).

Beyond such metabolic reprogramming, lactylation also directly supports CSC properties by stabilizing core stemness regulators and enhancing their functions. In CRC and esophageal squamous cell carcinoma (ESCC), lactylation of β-catenin enhances its protein stability and potentiates Wnt signaling activity, while lactylation of SHMT2 augments its enzymatic activity to promote serine metabolism, thereby providing nucleotide precursors essential for CSC self-renewal (50, 82). Substantially, these mechanisms, ranging from metabolic feedback loops to direct regulation of signaling pathways and metabolic enzymes, illustrate the multifaceted and hierarchical nature of lactylation in orchestrating CSC maintenance and therapy resistance.

Collectively, the mechanisms by which lactylation modification drives therapeutic resistance exhibit a clear hierarchical pattern. In the context of radiotherapy and chemotherapy, its role primarily converges on enhancing DNA damage repair capacity. In targeted therapy, the emphasis shifts toward remodeling oncogenic signaling pathways to sustain cell survival. Within the immunotherapy paradigm, the core mechanism pivots to regulating immune cell functions and sculpting a suppressive microenvironment. Underpinning all these therapeutic modalities, the maintenance of cancer stem cell traits constitutes the fundamental basis for the resistant phenotype. This progressive trajectory, extending from repair mechanisms through signaling and immune modulation to stemness profoundly illustrates the multifaceted adaptive involvement of lactylation modification as tumor cells confront therapeutic stress. To systematically present the landscape of lactylation-driven resistance mechanisms across different cancer types and treatment modalities, key evidence categorized by lactylation target/site, functional mechanism, and experimental model system is summarized (**Table 1**).

**Table 1:** Mechanisms of lactylation in cancer drug resistance.

Cancer type	Lactylationtarget/site	Function and mechanism	Experimental model and evidence sources
Glioblastoma	XRCC1 K247	Promotes DNA repair via nuclear translocation, conferring radiotherapy resistance (9).	Glioblastoma cell line and orthotopic xenotransplantation model
	H3K9	Induces mismatch repair deficiency via MLH1 intron retention, leading to temozolomide resistance (66).	
CRC	eEF1A2 K408	Enhances translation elongation and protein synthesis, driving tumor progression (56).	CRC cell lines and xenotransplantation model in mice
	H3K18	Activates autophagy via RUBCNL transcription, contributing to bevacizumab resistance (132).	CRC cell line and patient derived xenotransplantation model
	β-catenin	Stabilize proteins and activate Wnt pathways to promote dryness and drug tolerance (50).	CRC and ESCC cell lines
Bladder Cancer	H3K18	Activates transcription factors YY1/YBX1, conferring cisplatin resistance (69).	Bladder cancer cell lines and cisplatin resistant cell lines
Gastric Cancers	NBS1 K388	Enhances HR repair, leading to platinum/PARPi resistance (47).	Gastric cancer cell lines and patient derived organoids
Colorectal, Lung Cancers	MRE11 K673	Promotes HR via DNA binding, conferring platinum/PARPi resistance (48).	Multiple cancer cell lines, patient derived organoids and PDX models
Hepatocellular Carcinoma	CCNE2 K348	Accelerates cell cycle progression, driving tumor growth (101).	Hepatocellular carcinoma cell line and xenotransplantation model in mice
NSCLC	H3K18	Activates MYC/PD-L1 pathway via POM121, promoting immunotherapy resistance (77).	NSCLC cell line and immune perfect mouse model
	APOC2 K70	Induces Treg accumulation via lipolysis, contributing to immunotherapy resistance (108).	Immunohistochemical analysis of breast cancer cell lines and clinical specimens
Breast Cancer	H3K18	Forms feedback loop activating LDHA and PPARD, promoting glycolysis and survival (30).	PDAC cell line and its xenotransplantation model in mice
Multiple Cancers (Broad Spectrum)	Histones (e.g., H3K18, H3K9)	Alters chromatin state to transcribe resistance genes (e.g., ABC transporters, PD-L1), causing multi-drug resistance (99).	A variety of cancer cell lines and drug resistance models
	Non-histone substrates	Modulates protein stability, activity, and localization, contributing to multi-drug resistance (92).	A variety of cancer cell lines and functional verification experiments

## 4 Clinical translation strategies targeting the glycolysis-driven lactylation signaling network to overcome tumor therapy resistance

**Figure 2:**
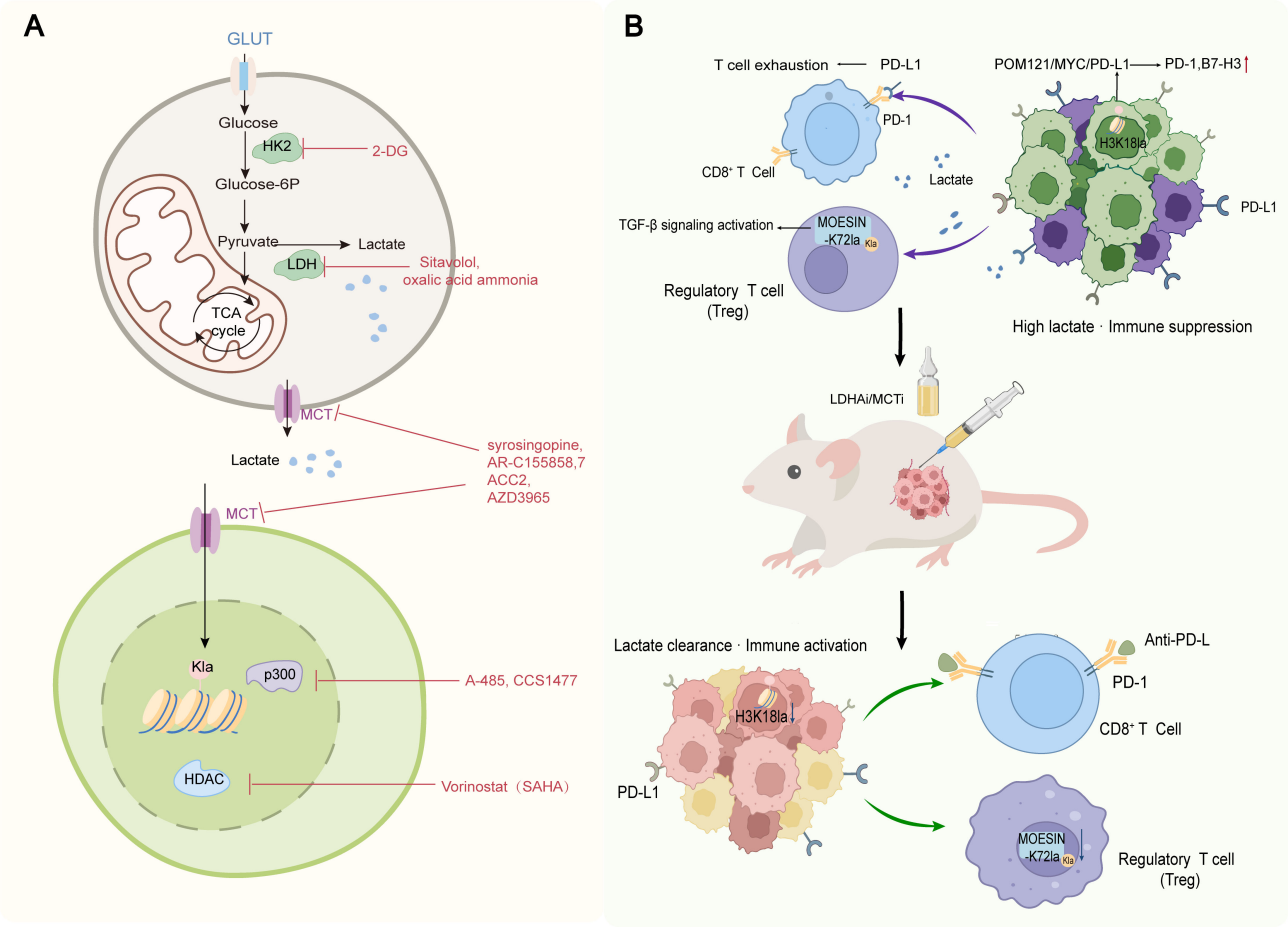
The strategy of targeting glycolysis lactylation axis to overcome the drug resistance of tumor therapy. **(A)** The intervention strategies of glycolysis and lactylation modification include HK, LDHA, p300/CBP, HDAC and MCT1/4 inhibitors. **(B)** The synergy between metabolic intervention and immunotherapy shows how reducing lactate levels can reverse immunosuppression and reactivate CD8^+^T cells. HK, hexokinase; Keywords lactate dehydrogenase A; MCT, monocarboxylate transporter; Regulatory T cells.

Here we summarize the therapeutic landscape of targeting the glycolysis-lactylation axis. Intervention strategies fall into three categories: upstream inhibition of glycolytic enzymes (e.g., LDHA inhibitors) and lactate transporters (MCT1/4 inhibitors), direct targeting of the lactylation machinery (e.g., p300/CBP inhibitors), and combination therapies that integrate these metabolic approaches with conventional treatments (**Figure 2A**). The clinical translation strategy targeting the glycolysis-lactylation axis follows a logical hierarchy progressing from upstream to downstream, and from metabolism to modification. Upstream interventions target lactate production (glycolytic enzymes) and transport (MCTs), aiming to block lactate accumulation and its driven modifications at the source. Downstream interventions directly target lactylation itself, including modification sites, writer enzymes, and eraser enzymes. This stratified framework provides a theoretical basis for evaluating the drug ability and clinical prospects of different targets.

### 4.1 Strategies targeting the glycolytic pathway

#### 4.1.1 Inhibitors of key glycolytic enzymes

Targeting core enzymes of the glycolytic pathway is a direct strategy to intervene in this metabolic axis. High LDHA expression correlates with poor patient prognosis in various cancers, including liver cancer, lung cancer, and pancreatic cancer (9). The therapeutic efficacy of the LDHA inhibitor sodium oxamate has been validated in humanized mouse models of non-small cell lung cancer(NSCLC), this inhibitor not only directly suppresses tumor growth but also increases intratumoral CD8^+^ T cell infiltration by approximately 2- to 3-fold, exerting synergistic antitumor effects when combined with PD-1 inhibitors (83, 84). By inhibiting lactate production, this strategy effectively remodels the immunosuppressive microenvironment, converting immunologically “cold” tumors into “hot” tumors to ultimately exert antitumor effects (84, 85). Similarly, in melanoma models with LDHA deficiency, enhanced infiltration of NK cells and CD8^+^ T cells, as well as increased sensitivity to PD-1 inhibitors, has been observed (86). Furthermore, using siRNA to target LDHA can halt tumor progression by inducing oxidative stress and cell death (87).

The hexokinase inhibitor 2-deoxy-D-glucose (2-DG) blocks glycolysis and induces apoptosis by competitively inhibiting hexokinase (88). Currently, 2-DG has entered Phase I clinical trials, demonstrated a tolerable safety profile in patients with advanced solid tumors when combined with docetaxel (89). Additionally, 2-DG has been shown to enhance the radiosensitivity of prostate cancer, pancreatic cancer, and other cells by reducing glutathione levels or activating AMPK-induced autophagy (90, 91).

#### 4.1.2 Targeting the lactate transport system

MCT1 and MCT4, which mediate lactate efflux into the tumor microenvironment (TME), represent key targets for regulating TME acidity (92). Inhibition of MCT4, a high-affinity lactate transporter, has been shown to potentiate the antitumor efficacy of anti-PD-1 therapy via both genetic and pharmacological approaches (93, 94). MCT4 is highly expressed in tumors such as hepatocellular carcinoma and is associated with poor response to anti-PD-1 treatment (74). Fang et al. demonstrated that genetic or pharmacological inhibition of MCT4 with the highly effective inhibitor VB124, in immunocompetent Hepatocellular Carcinoma mouse models, promoted CD8^+^ T cell infiltration and cytotoxic function, effectively suppressing tumor progression (93). Although numerous MCT inhibitors, including syrosingopine, AR-C155858, 7ACC2, and AZD3965 have been developed in preclinical studies, only the MCT1 inhibitor AZD3965 has completed a Phase I dose-escalation trial (NCT01791595) in patients with advanced solid tumors and lymphoma. The results confirmed its acceptable safety profile; however, its single-agent antitumor activity was limited, suggesting that this class of agents is more likely to realize its clinical value in combination therapeutic strategies (95). Preclinical evidence indicates that AZD3965 in combination with anti-PD-L1 therapy reduces lactate secretion in the TME and reverses T cell exhaustion (46). Notably, MCT4 inhibition and MCT1 inhibition may produce differential effects when combined with immunotherapy, stemming from their distinct cellular expression profiles: MCT4 is primarily expressed in glycolysis-dependent tumor cells, mediating lactate efflux, whereas MCT1 is also highly expressed in various immune cells, such as T cells, where it participates in metabolic adaptation (96). Therefore, target selection requires comprehensive consideration of tumor-specific characteristics and preservation of immune cell function.

However, systemic inhibition of glycolysis or lactate transport may trigger off-target effects, such as pyruvate accumulation or disruption of immune homeostasis (97). Furthermore, compared to directly targeting LDHA, metabolic interventions aimed at upstream glycolytic regulators may elicit broader biological effects and potential unpredictability (8). Therefore, optimizing dosing regimens and precisely identifying beneficiary populations are key to realizing their clinical potential.

The aforementioned upstream intervention strategies targeting lactate production and transport offer the advantage of simultaneously reversing microenvironmental acidification and inhibiting lactylation modifications, thereby achieving dual antitumor effects. However, due to the basal expression of glycolytic enzymes and MCTs in normal tissues, such strategies face challenges regarding their therapeutic window. In contrast, directly targeting downstream lactylation modifications themselves holds promise for achieving more precise intervention. This, however, necessitates a deeper understanding of the regulatory mechanisms governing these modifications.

### 4.2 Targeting lactylation modification

#### 4.2.1 Writer inhibitors

The histone acetyltransferase p300 has been demonstrated to possess lactyltransferase activity, capable of specifically catalyzing lactylation modifications at sites such as histone H3K9 and H3K18. Furthermore, high p300 expression is associated with poor prognosis in various malignancies, including liver cancer, prostate cancer, and lymphoma (98). In multiple cancer cell lines and xenograft models, the small-molecule inhibitors of CBP/p300, A-485 and CCS1477, specifically suppress lactylation at key gene loci such as PD-L1 and vascular cell adhesion molecule 1, thereby reversing chemotherapy resistance and inhibiting epithelial-mesenchymal transition (99). Currently, CCS1477, as the first p300/CBP inhibitor to enter clinical development, is undergoing phase I/IIa trials in patients with advanced hematologic malignancies and solid tumors such as castration-resistant prostate cancer (NCT03568656) (98).

Furthermore, Zong et al. demonstrated that the function of alanyl-tRNA synthetase (AARS1) as a non-canonical lactylation writer can be inhibited by β-alanine. In animal models, β-alanine blocks the binding of lactate to AARS1, thereby suppressing p53 lactylation-dependent functional inactivation and significantly inhibiting tumorigenesis (57).

#### 4.2.2 Eraser activators

In contrast to inhibiting writer enzymes, activating eraser enzymes represents an alternative approach for modulating lactylation levels. By enhancing delactylation activity, the levels of pathogenic lactylation modifications can likewise be reduced. Sirtuin-activating compounds (STACs), including natural activators such as honokiol and resveratrol, as well as synthetic small molecules like SRT1720 and SRT1460, have demonstrated potential in modulating this pathway (100). In a mouse hepatocellular carcinoma model, honokiol activated SIRT3, leading to the delactylation of the cell cycle protein CCNE2, consequently inducing apoptosis of liver cancer cells and inhibiting tumor proliferation (101). However, although these compounds show promise in preclinical studies, their systemic toxicity and appropriate dosing regimens require further evaluation (102). The HDACi Vorinostat (SAHA), although approved for lymphoma treatment, requires further exploration regarding its regulatory role on lactylation modification and its potential for combination therapy in solid tumors (103).

The SIRT family (NAD^+^-dependent deacetylases) plays significant roles in tumor suppression. Experimental studies by Jin and Zu confirmed that SIRT2 and SIRT3 can catalyze delactylation of non-histone proteins, thereby inhibiting tumor progression (40, 101). Although no eraser enzyme highly specific for lactylation modification has been identified to date, existing evidence suggests that deacetylases possess the potential to remove such modifications (104). Currently, all intervention strategies targeting lactylation-related enzymes are in the preclinical or early clinical stages. The primary challenge lies in the broad functional involvement of these enzymes in physiological processes, posing a high risk of off-target effects.

#### 4.2.3 Targeting lactylation modification sites

In contrast to the aforementioned strategies that indirectly modulate lactylation levels by regulating enzyme activity, a more precise interventional approach involves directly targeting the lactylation modification sites themselves, either by recognizing or blocking pathogenic modification events with specific antibodies, including pan-specific antibodies (anti-pan-Kla) that can identify lactic acid lysine residues in all proteins, and multi-site specific antibodies, such as anti-H3K18la, anti-H3K14la and anti-XRCC1 K247la. Their utility has been validated in various tumor models, including glioblastoma, bladder cancer, and melanoma, providing crucial tools for exploring lactate-driven tumor epigenetic regulation (105–107).

Targeting lactylation epitopes in combination with existing immunotherapies may hold clinical translation potential. In NSCLC, tumor cells accumulate lactate through glycolysis, which is subsequently utilized by p300 to catalyze lactylation of apolipoprotein C-II (APOC2) at lysine 70. This modification enhances APOC2 stability by inhibiting its ubiquitin-proteasome degradation. Stabilized APOC2 subsequently binds to lipoprotein lipase, promoting the hydrolysis of triglycerides within the tumor microenvironment and releasing substantial quantities of free fatty acids. The enrichment of extracellular free fatty acids provides a selective metabolic advantage for the survival and expansion of Tregs within the glucose-deprived microenvironment, leading to increased Treg infiltration, suppression of CD8^+^ T cell function, and ultimately driving resistance to PD-1 antibody immune therapy (108). Notably, the APOC2 K70 lactylation-specific antibody developed by Chen et al. was applied to a clinical specimen cohort comprising matched samples obtained before treatment and after treatment failure, revealing that its immunohistochemical reactivity was positively correlated with resistance to immunotherapy in patients with NSCLC (108). However, research on therapeutic antibodies targeting histone lactylation sites has not yet achieved a breakthrough. The primary obstacle lies in achieving selective inhibition of histone lactylation without disrupting normal cellular functions (109). Furthermore, the potential off-target effects of broad-spectrum histone lactylation inhibitors and their impact on other cellular functions remain to be clarified.

### 4.3 Combination therapeutic strategies

The mechanistic rationale for combining metabolic interventions with immunotherapy lies in the dual effect of LDHA or MCT inhibition: reducing lactate levels not only suppresses lactylation-driven resistance but also reverses immunosuppression by reactivating CD8^+^ T cell function and attenuating Treg accumulation (**Figure 2B**). Harnessing such multi-mechanistic synergism through the integration of metabolic and conventional therapies represents a pivotal strategy for overcoming drug resistance.

#### 4.3.1 Metabolic intervention + radiotherapy

As previously discussed, intratumoral lactate accumulation drives radioresistance by enhancing DNA repair capacity and alleviating oxidative stress. Yang et al. demonstrated that combining an LDHA inhibitor with radiotherapy significantly sensitizes prostate cancer cells to radiation. This effect involves depletion of intracellular ATP, promotion of reactive oxygen species accumulation, and impairment of DNA damage repair capability (110). Furthermore, statins such as fluvastatin effectively inhibit MCT4 function. In lung adenocarcinoma models, disrupting lactate export homeostasis through MCT4 inhibition induces metabolic stress, thereby enhancing radiotherapy efficacy (99). Collectively, these findings indicate that targeting lactate metabolism represents a viable radiosensitization strategy.

#### 4.3.2 Metabolic intervention + chemotherapy

Key glycolytic molecules have emerged as novel targets for chemotherapy sensitization. Weng et al. discovered that a novel GLUT1 inhibitor, when combined with cisplatin, produces synergistic antitumor effects in breast cancer models through suppression of the Akt/mTOR signaling pathway (111). Furthermore, research by KORGA et al. confirmed the synergistic efficacy of glycolytic inhibitors with doxorubicin in hepatocellular carcinoma treatment. This combination strategy effectively reduces cell viability and induces apoptosis, thereby further corroborating the feasibility of integrating metabolic intervention with conventional chemotherapy (112).

#### 4.3.3 Metabolic intervention + targeted therapy

Directly targeting lactylation modification can effectively reverse resistance to targeted therapy. The anti-epileptic drug stiripentol has been demonstrated to inhibit lactylation of the NBS1 protein, impairing DNA damage repair capacity. Simultaneously, it reduces lactylation levels by inhibiting LDHA/B activity, significantly enhancing the sensitivity of glioblastoma to temozolomide (47, 66). Moreover, Lu et al. revealed that enhanced glycolysis drives lenvatinib resistance in hepatocellular carcinoma by inducing lactylation of Insulin-like growth factor 2 mRNA binding proteins (IGF2BP1, IGF2BP2 and IGF2BP3). Inhibition of this pathway using IGF2BP3 siRNA or 2-DG successfully reversed such resistance (113), demonstrated that targeting the lactylation-metabolic axis represents an effective strategy to overcome resistance to targeted therapies.

#### 4.3.4 Metabolic intervention + immunotherapy

The central role of lactylation modification in forming the immunosuppressive microenvironment makes it an ideal target for improving immunotherapy response (114). The combination of LDHA inhibitors with PD-1 blockers significantly inhibits lactate production and downstream lactylation modifications, synergistically enhancing anti-tumor immune activity (46). Sun et al. found that oxalate, a lactic acid production inhibitor, can enhance the therapeutic effect of CAR-T cells in glioblastoma by regulating the immunomolecular phenotype (115). Furthermore, the natural bioactive compound evodiamine suppresses HIF1α-associated H3K18 lactylation, thereby downregulating the expression of semaphoring 3A (Sema3A) (116). Sema3A exerts dual functions within the tumor microenvironment by promoting vascular normalization and modulating immune cell trafficking; its downregulation contributes to ameliorating tumor hypoxia and reversing immunosuppressive states. Concurrently, evodiamine inhibits PD-L1 expression and induces ferroptosis, thereby synergistically enhancing immunotherapy responses through a multidimensional metabolic-epigenetic-immune mechanism (116). However, research by Yu’s team indicates that Evodiamine may exhibit hepatotoxicity, though definitive dose-toxicity relationship studies remain lacking (116).

#### 4.3.5 Emerging combination strategies

Drug delivery systems based on nanotechnology offer new avenues for optimizing metabolic interventions. Nanocarrier systems effectively overcome the limitations of traditional lactate inhibitors, such as poor stability and limited targeting specificity, by enhancing targeted delivery efficiency and intratumoral retention of drugs (117). PLGA nanoparticles loaded with 2-DG can significantly reduce intratumoral lactate levels and enhance T cell-mediated anti-tumor immunity (118). Additionally, a pH-responsive AZD3965 nano-formulation enables specific drug release in the acidic tumor microenvironment. When combined with anti-PD-1 therapy, it significantly reduces the required dosage while improving the survival rate in model animals (119).

Furthermore, the cross-regulation between lactylation modification and other cell death modalities shows potential. Cuproptosis is a novel form of regulated cell death induced by mitochondrial Cu²^+^ overload, dependent on the aggregation of lipoylated tricarboxylic acid cycle proteins and the destabilization of iron-sulfur cluster proteins (120). In gastric cancer, copper toxic stress induces lactylation of the METTL16 protein, subsequently enhancing sensitivity to cuproptosis. This process can be further amplified by the SIRT2 inhibitor AGK2, providing a theoretical basis for developing novel combination strategies (121).

Based on the above discussion, targeting glycolytic enzymes, lactic acid transport protein or lactic acid modifier enzyme lines upstream on this axis and using them with conventional therapy shows a broad prospect of reverse drug resistance.

The aforementioned combination strategies, based on their synergistic mechanisms, can be categorized into three paradigms characterized by direct sensitization, signaling synergy, and immune remodeling. The commonality underlying these three paradigms consistently indicates that the optimal clinical application strategy for targeting the glycolysis-lactylation axis lies not in single-agent intervention, but rather in its organic integration with existing therapeutic modalities to achieve synergistic enhancement. A comprehensive overview of representative combination strategies targeting this axis, organized by combination modality and accompanied by their mechanisms of action and experimental models, is provided (**Table 2**).

**Table 2:** Combined therapeutic strategies targeting the glycolysis-lactylation axis.

Combination strategy	Representative agents	Mechanism of action	Model and efficacy
Metabolic Intervention + Radiotherapy	LDHA inhibitor (e.g., Stiripentol)	Induces ATP depletion and ROS accumulation, impairing DNA repair.	Enhances radiosensitivity in prostate cancer (110).
	Fluvastatin (MCT4 inhibitor)	Disrupts lactate export, induces metabolic stress	Enhances radiotherapy efficacy in lung adenocarcinoma (99).
Metabolic Intervention + Targeted Therapy	Stiripentol + Temozolomide	Inhibits LDHA/B & NBS1 lactylation, impairs DNA repair	Restores TMZ sensitivity in glioblastoma (47).
	Liposomal IGF2BP3 siRNA/2-DG + Lenvatinib	Inhibits IGF2BP2 lactylation, reversing metabolic reprogramming.	Restores lenvatinib sensitivity in hepatocellular carcinoma (54).
Metabolic Intervention + Immunotherapy	LDHA inhibitor + anti-PD-L1	Reduces lactate, remodels TME, reverses T cell exhaustion.	Synergistic tumor suppression in NSCLC models (46).
	pH-responsive AZD3965 nanodrug + anti-PD-L1	Enhances targeting, reduces toxicity, synergistically modulates TME	Improves efficacy and survival in mouse models (119).
Emerging Strategies	SIRT2 inhibitor (AGK2) + Copper ionophore	Inhibits METTL16 lactylation, enhancing cuproptosis sensitivity.	Synergistic effect in gastric cancer models (121).

## 5 Challenges and future perspectives: from mechanistic complexity to precision intervention

The elucidation of the glycolysis-lactylation axis has fundamentally redefined our understanding of therapy resistance, yet its clinical translation is confronted by a series of interconnected challenges that span from fundamental biology to therapeutic application. Addressing these obstacles requires a shift in perspective: from viewing lactylation as an isolated modification to recognizing it as a node within a highly complex and adaptive network. Below, we dissect these challenges, offering our perspective on the critical bottlenecks and potential paths forward.

### 5.1 The unresolved complexity of the lactylation network

While the core writer-eraser-reader enzymes for lactylation are being identified, our understanding remains fundamentally incomplete a primary obstacle to rational therapeutic design. First, the full spectrum and substrate specificity of these regulators are not fully characterized, leaving open critical questions about how specific lactylation events are orchestrated in different cellular contexts (30, 122). Second, and more critically, lactylation does not operate in isolation. It is embedded within a dense network of metabolism-derived PTM, including acetylation and succinylation, which often compete for identical lysine residues and can exert opposing functional effects (122). The rules governing this competitive crosstalk remain largely unexplored, yet they have profound implications: a pan-enzyme inhibitor targeting a writer like p300 or an eraser like HDAC would indiscriminately disrupt the entire acylation landscape, potentially leading to unpredictable on-target toxicities and a narrow therapeutic window (5, 59, 123–125). This knowledge deficit directly impedes the development of highly selective pharmacological tools, suggesting that future efforts must prioritize dissecting this complex PTM code at single-site resolution before effective interventions can be realized.

### 5.2 The challenge of metabolic and functional plasticity

Beyond the complexity of the modification itself, the adaptive nature of the tumor system presents another formidable barrier. Tumor cells exhibit remarkable metabolic plasticity, when glycolysis is therapeutically inhibited, they can rewire alternative pathways such as glutaminolysis, lipid metabolism, and nucleotide synthesis to sustain survival and redox balance (126, 127). This compensatory network activation suggests that single-node interventions targeting lactate production alone are likely to be circumvented. Furthermore, this plasticity extends to the functional roles of lactylation itself. As discussed, lactylation can drive both pro-tumorigenic (e.g., DNA repair, immunosuppression) and, under certain contexts, potential anti-tumorigenic effects (49, 76, 77, 101). This dualism complicates therapeutic strategies, as globally inhibiting lactylation might inadvertently suppress beneficial immune responses while aiming to block resistance mechanisms. A key future challenge, therefore, is to understand how the context dictated by cell type, metabolic state, and microenvironmental signals determines the functional outcome of lactylation, enabling the design of interventions that selectively target its pathogenic roles.

### 5.3 Tumor heterogeneity and the need for spatiotemporal resolution

The clinical translation of lactylation-based strategies is further confounded by profound tumor heterogeneity. Lactylation landscapes are not static or uniform, they exhibit significant spatiotemporal variability across different tumor types, distinct microregions within the same tumor (e.g., hypoxic vs. oxygenated niches), and throughout the course of treatment under therapeutic pressure (128). Conventional bulk-tissue analyses provide only a static, population-averaged snapshot, obscuring the dynamic, clone-specific modification events that likely drive the evolution of acquired resistance (129). This heterogeneity poses a dual challenge: it limits the utility of lactylation as a reliable predictive biomarker and renders therapeutic strategies that target a single, uniformly expressed node susceptible to failure (19, 69). Overcoming this requires a paradigm shift towards high-resolution analysis. The integration of single-cell and spatial multi-omics technologies is not merely a technical advance but a conceptual necessity to map the cellular ecosystems of resistance and identify the dominant subpopulations and their regulatory hubs (129–131).

### 5.4 From correlation to causation: a critical roadblock

A final, overarching challenge is the field’s heavy reliance on correlational data. Numerous studies link lactylation levels to prognosis or resistance, but establishing a causal, mechanistic role for specific lactylation events remains the exception rather than the rule (8, 42, 48). This gap between correlation and causation is a critical roadblock to translation, as it prevents the prioritization of true driver events for therapeutic targeting (9, 54). As highlighted by the elegant work of Chen et al. on MRE11 K673 lactylation, the path forward requires a systematic approach: integrating unbiased lactylome profiling with functional genomic screens (e.g., CRISPR-Cas9) to identify candidate resistance drivers, followed by rigorous causal validation using site-specific mutagenesis in physiologically relevant models such as patient-derived organoids and humanized mice (48, 84, 93). This transition from descriptive mapping to functional interrogation is essential to build a robust pipeline for translating lactylation discoveries into actionable therapeutic targets.

## 6 Conclusion

In conclusion, lactylation modification represents a fundamental mechanistic bridge coupling tumor metabolic reprogramming with epigenetic regulation, providing a conceptual framework for understanding broad-spectrum therapy resistance in cancer. By integrating hallmark cancer traits, including metabolic alteration, genomic instability, and immunosuppression the glycolysis-lactylation axis emerges as a high-value therapeutic node. Representative strategies targeting this axis, from glycolytic enzyme inhibitors to lactylation modulators, have advanced from preclinical validation to early-phase clinical trials, with select agents demonstrated preliminary safety and target engagement. However, significant challenges remain, including incomplete mechanistic understanding, metabolic plasticity, tumor heterogeneity, and the need for causal validation. Addressing these obstacles requires a systematic trajectory, including multi-omics dissection at single-cell resolution, rigorous causal validation in physiologically relevant models, and integration of multidimensional datasets through artificial intelligence to construct predictive models for patient stratification and combination therapy optimization. Through this integrated approach, the glycolysis-lactylation axis can be translated from a biological concept into a therapeutically actionable framework, ultimately offering new strategies to overcome the enduring challenge of therapy resistance in cancer.
